# Gas hydrate saturations estimated from pore-and fracture-filling gas hydrate reservoirs in the Qilian Mountain permafrost, China

**DOI:** 10.1038/s41598-017-16531-x

**Published:** 2017-11-24

**Authors:** Kun Xiao, Changchun Zou, Zhenquan Lu, Juzhi Deng

**Affiliations:** 1 Fundamental Science on Radioactive Geology and Exploration Technology Laboratory, East China University of Technology, Nanchang, People’s Republic of China; 2 School of Geophysics and Information Technology, China University of Geosciences (Beijing), Beijing, People’s Republic of China; 3 Oil and Gas Survey, China Geological Survey, Beijing, People’s Republic of China

## Abstract

Accurate calculation of gas hydrate saturation is an important aspect of gas hydrate resource evaluation. The effective medium theory (EMT model), the velocity model based on two-phase medium theory (TPT model), and the two component laminated media model (TCLM model), are adopted to investigate the characteristics of acoustic velocity and gas hydrate saturation of pore- and fracture-filling reservoirs in the Qilian Mountain permafrost, China. The compressional wave (P-wave) velocity simulated by the EMT model is more consistent with actual log data than the TPT model in the pore-filling reservoir. The range of the gas hydrate saturation of the typical pore-filling reservoir in hole DKXX-13 is 13.0~85.0%, and the average value of the gas hydrate saturation is 61.9%, which is in accordance with the results by the standard Archie equation and actual core test. The P-wave phase velocity simulated by the TCLM model can be transformed directly into the P-wave transverse velocity in a fracture-filling reservoir. The range of the gas hydrate saturation of the typical fracture-filling reservoir in hole DKXX-19 is 14.1~89.9%, and the average value of the gas hydrate saturation is 69.4%, which is in accordance with actual core test results.

## Introduction

Gas hydrate is a solid crystal with a cage structure consisting of water molecules and natural gas (mainly CH_4_), which mainly exists in the terrestrial permafrost regions and beneath the sea along the outer continental margins of the world’s oceans^1^. As a new clean alternative energy source with huge reserves, gas hydrates have attracted more and more attention, and more than 100 regions have directly or indirectly been found to have gas hydrate occurrences^2,3^. In November, 2008, gas hydrate samples were recovered in the Qilian Mountain permafrost (QMP), Qinghai Province of China^3,4^. This is the first discovery of gas hydrate in China’s permafrost and occurs in the global low-middle latitude permafrost zone. The QMP may be the most promising strategic area for gas hydrate^4,5^.

In recent years, in order to further delineate the promising areas of gas hydrate, and to provide practical technical support for exploration and development of gas hydrate, 2D seismic reflection and comprehensive well log projects have been launched. However, because research on the gas hydrate in the permafrost region in our country has been at its very early stage, related geophysical exploration methods are rarely used to prospect for gas hydrates. At present, there are few studies on the properties of gas hydrate reservoirs and the evaluation of gas hydrate saturation for the permafrost zone, and few related reports come into sight. Accurate evaluation of gas hydrate resources are directly related to the understanding of the value of gas hydrate resources, their environmental impact, and any potential hazards^2,6^. Therefore, the evaluation of gas hydrate saturation is important in the exploration for and development of gas hydrate resources^7,8^. Geophysical well logging is the most direct method to obtain the reservoir parameters of gas hydrate *in situ*, and also to calculate the gas hydrate saturation^9^. Amongst the geophysical well logging tools used to evaluate gas hydrate saturation, the acoustic log is one obvious logging parameter for the gas hydrate reservoir that has been widely used in research on gas hydrate saturation^10–15^.

In the sample of gas hydrate from the QMP, there are two obvious different filling patterns: pore-filling and fracture-filling^3,4^. The gas hydrate displaces the fluid within the pores of the rock matrix in the pore space and is indistinguishable to the naked eye. The gas hydrate that is produced in the cracks does not occupy the pore space, but forces the formation rock to open and form cracks and fill them with the visible white ice layer^14,16^. For the different types of gas hydrate reservoir, adopting a velocity model of an isotropic porous medium for the inversion of gas hydrate saturation will lead to large errors in the resource estimations. Logging must usefully identify gas hydrate reservoirs and calculate gas hydrate saturation, and also to provide a reference for regional gas hydrate evaluation. To do this, an analysis of the acoustic velocity characteristics must be made for the gas hydrate saturation estimation within the different types of reservoirs present in the study area.

In this paper, the reservoir characteristics of gas hydrate in the study area are analyzed using ultrasonic imaging logging and drilling core data. The acoustic velocity characteristics of pore filling gas hydrate reservoir in the QMP are simulated using the forward modeling velocity model that is established by the effective medium theory (EMT) model, and the simulation results are compared with the elastic wave velocity model based on the two-phase medium theory (TPT) model. The acoustic velocity characteristics of fracture-filling gas hydrate reservoir in the QMP are also simulated using the two component laminated media model (TCLM). Finally, the acoustic velocities that are simulated by the pore- and fracture-filling reservoirs are used to estimate the gas hydrate saturation in the study area.

## Geological characteristics of gas hydrate

**Figure 1:**
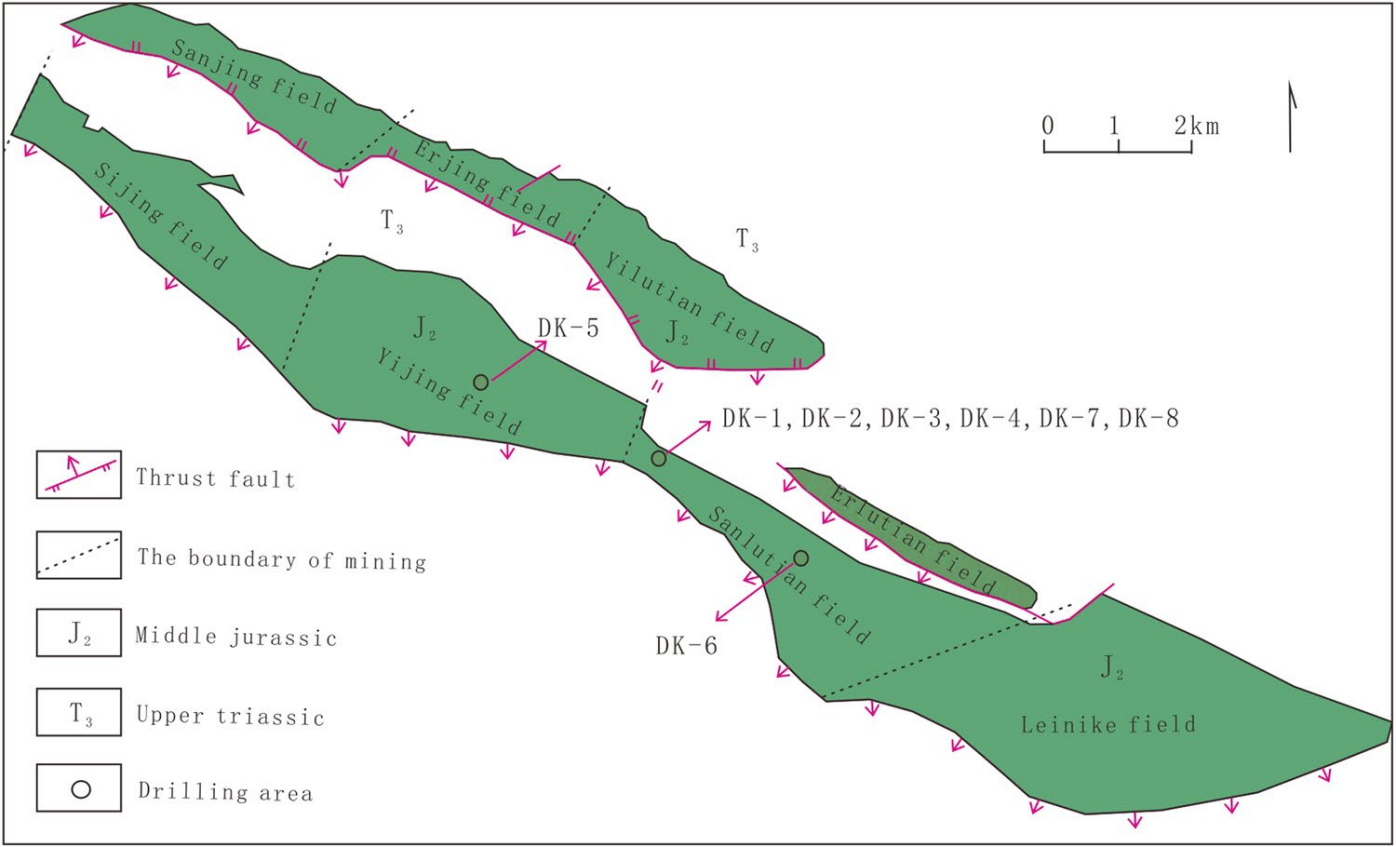
Locations of drilling sites of the scientific drilling project for gas hydrate in Qilian Mountain permafrost^4^. (The figure is generated by CorelDRAW ×6 software by Corel Corporation, and the company website address is http://www.corel.com/cn/).

The Qilian Mountain permafrost is located on the northern margin of the Tibetan Plateau, and the permafrost area is about 10 × 10^4^ km^2^. In recent years, the gas hydrate samples are obtained firstly by drilling in the Juhugeng mining of the Muli coal field^3,4^. The drilling area is tectonically situated in the western Middle Qilian block formed during the Caledonian Movement, adjacent to the South Qilian structural zone. Except for the Quaternary deposits, the exposure strata mainly include Jiangcang Formation (J2j) and Muli Formation (J2m) of middle Jurassic age. The lithology of the drilling formation is mainly mudstone, siltstone, fine sandstone, medium sandstone, and coarse sandstone. Drilling test wells of DK-1, DK-2, Dk-3, DK-4, DK-5, DK-6, DK-7 and DK-8 were carried out, and the gas hydrate was acquired in multiple layers of the wells DK-1, DK-2, DK-3, DK-7 and DK-8 (Fig. 1). Gas hydrate was mainly found in the Jiangcang Formation of the middle Jurassic, and occurred within intervals of about 133–396 meters below the surface, below the permafrost in the drilling area.

The combined information from drilling and core experiment studies shows that gas hydrates mainly occur in the Jiangcang Formation of Middle Jurassic in the Muli permafrost. The geological and occurrence characteristics indicate that gas hydrates occur in separate reservoirs below the base of permafrost. In addition, gas hydrates are vertically distributed non-continuously in each borehole, and the nature of the gas hydrate distribution in the lateral areas between drill holes is not apparent due to the rock fracture system that plays an important role in gas hydrate distribution^5^.

Specific geological characteristics of the gas hydrate reservoirs are summarized in Table 1. Compared to gas hydrate samples from the Mackenzie Delta of Canada, gas hydrate in the QMP can be regarded as a new type gas hydrate characterized by a relatively shallow buried depth, thin thickness of permafrost, complex gas components, and coal-bed methane gas source, which is of scientific, economic, and environmental significance^4^.

**Table 1:** Comparison of formation properties between the Qilian Mountain and Mackenzie Delta gas hydrates.

District	Mackenzie Delta^49,50^	Qilian Mountain^3,4^
Property		
Gas component	CH_4_	CH_4_, C_2_H_6_, C_3_H_8_, C_4_H_10_, CO_2_
Structure type	sI	sII
Stratum	Oligocene (Kugmallit, Mackenzie Bay)	Middle Jurassic (Jiangcang)
Reservoir lithology	Sandstone	Oily shale, siltstone, fine sandstone
Permafrost	Arctic	Mountain
Permafrost thickness	640 m	95 m
Burial depth	>1000 m	133~396 m

## Numerical simulation methods

When the lithology of the gas hydrate reservoir matrix is sandstone and formation fracture development is small, the gas hydrate will be disseminated to fill the pores of the sandstone^17,18^. In previous studies of the acoustic velocity characteristics of pore-filling gas hydrate reservoirs, marine gas hydrates have been studied in the early stages of formation. The effective medium theory, contact media theory, and relevant theory of porous media were used to establish the different forward modeling velocity models, included the EMT model and the TPT model^19–23^. In order to associate the microscopic distribution form with the physical parameters of gas hydrate formation, the elastic wave velocity model based on effective medium theory can be used^24^. The model is established based on the effective medium theory, considering the rock mechanics of elasticity and statistics; the combination of the macroscopic and microscopic aspects of wave propagation in the medium has a relatively wide range of use. In this paper, the EMT model is used to carry out the research work on the pore-filling gas hydrate reservoir in the QMP.

Because there are many parameters that must be set in the simulation of elastic wave velocity using the TPT model, there are some limitations in practical application^22,25^. In order to verify the accuracy of simulation results by the EMT model, the TPT model is selected for comparison study.

When the cracks of gas hydrate reservoir are more developed, the gas hydrates usually fill the cracks in the formation rocks with meshes, nodules, or veins^3,26^. Because the dips of the cracks in the gas hydrate reservoir are generally relatively steep, and the distributions of cracks are often controlled by the regional tectonic principal stresses, the cracks will in general be directionally aligned, leading to anisotropic characteristics in the gas hydrate occurrence zones^27^. If we were to assume that the gas hydrate filled the pores in isotropic rock, then the velocities of the forward modeling used for gas hydrate saturation inversion would yield large errors^26,28^.

For the stratigraphic anisotropy caused by directional alignment of the cracks, many previous studies have been carried out, and a variety of simplified models are proposed, including a laminated media model^29^, a media model where cracks are embedded in the pores^30,31^, periodic thin interlayers and expansion model^32^, and so on. For the fracture-filling gas hydrate reservoir, some scholars have used the TCLM model to carry out the acoustic velocity simulation and gas hydrate saturation estimation study in several gas hydrate potential areas, and have obtained the better application effect^26,33,34^. Therefore, this paper also selects the TCLM model to carry out related research work on the fracture-filling gas hydrate reservoir in the QMP.

### Numerical simulation of pore filling reservoir

#### Acoustic velocity forward simulation

In the actual use of the model, the gas hydrate reservoir can be regarded as the equivalent homogeneous medium, where microscopic distribution patterns affect the elastic parameters, and we study the characteristics of the elastic wave. According to the effective medium theory, the P-wave and S-wave velocities of gas hydrate formations are as follows:

1 $${V}_{p}=\sqrt{({K}_{{\rm{sat}}}+4/3{\mu }_{{\rm{sat}}})/{\rho }_{b}}$$

2 $${V}_{s}=\sqrt{{\mu }_{{\rm{sat}}}/{\rho }_{{\rm{b}}}}$$

where *V*_p_ is the P-wave velocity, *V*_s_ is the S-wave velocity, *K*_sat_ is the bulk modulus of the fluid-saturated rock formation, μ_sat_ is the shear modulus of fluid-saturated rock formation, and ${\rho }_{{\rm{b}}}$ is the density of the rock formation.

In order to determine the physical parameters (*V*_p_, *V*_s_, ${\rho }_{{\rm{b}}}$) of the gas hydrate reservoir in the study area, we need to determine the ${K}_{{\rm{sat}}}$ and ${\mu }_{{\rm{sat}}}$. The calculation of the two modulus parameters is referred to as the EMT model by Helgerud *et al*.^24^. The model considers the impacts of the formation physical parameters changing by mineral constituent, porosity, and gas hydrate saturation of the formation rock, and can be used to simulate the change characteristics of the gas hydrate reservoir in the QMP.

In order to compare the forward simulation results with the TPT model, the TPT model by Domenico^35^ is selected to carry out the relevant forward simulation work. The P-wave and S-wave velocities of the TPT model are as follows:

3 $${V}_{{\rm{p}}}={\{[(\frac{1}{{C}_{{\rm{m}}}}+\frac{4}{3}\mu )+\frac{\frac{{\varphi }_{{\rm{eff}}}}{k}\frac{{\rho }_{{\rm{m}}}}{{\rho }_{f}}+(1-\beta -2\frac{{\varphi }_{{\rm{eff}}}}{k})(1-\beta )}{(1-{\varphi }_{{\rm{ef}}f}-\beta ){C}_{{\rm{b}}}+{\varphi }_{{\rm{eff}}}{C}_{{\rm{f}}}}]\cdot \frac{1}{{\rho }_{{\rm{m}}}(1-\frac{{\varphi }_{{\rm{eff}}}}{k}\frac{{\rho }_{{\rm{f}}}}{{\rho }_{{\rm{m}}}})}\}}^{1/2}$$

4 $${V}_{{\rm{s}}}={[\frac{\mu }{{\rho }_{{\rm{m}}}(1-\frac{{\varphi }_{{\rm{eff}}}}{k}\frac{{\rho }_{{\rm{f}}}}{{\rho }_{{\rm{m}}}})}]}^{1/2}$$

where *V*_p_ is the P-wave velocity, *V*_s_ is the S-wave velocity, ${\varphi }_{eff}$ is effective porosity, *μ* is the average rigidity of the matrix, ${\rho }_{m}$ is the average density, ${\rho }_{f}$ is the density of the fluid phase, *β* is the proportionality coefficient, *k* is the coupling factor, and ${C}_{b}$, ${C}_{f}$, and ${C}_{m}$ are the compressibilities of the solid phase, the fluid phase, and the matrix, respectively. The use of equations (3) and (4) for computing the P-wave and S-wave velocities are as reported in Tinivella^22^.

#### Gas hydrate saturation inversion

When the acoustic velocity obtained from the forward modeling is used for inversion for the gas hydrate saturation, we need to compare the results with the actual borehole acoustic logging in the study area. The difference between the actual acoustic velocity of the gas hydrate reservoir and the acoustic velocity as simulated using the water-saturated pore space reflects the saturation of gas hydrate. The actual P velocity data of the gas hydrate reservoir can only be obtained relative to the normal acoustic travel time logging in the gas hydrate drilling borehole of QMP. So this research paper on gas hydrate saturation inversion of pore-filling reservoir will only be based on the P-wave velocity obtained from the EMT model used in the inversion to obtain the gas hydrate saturation.

**Figure 2:**
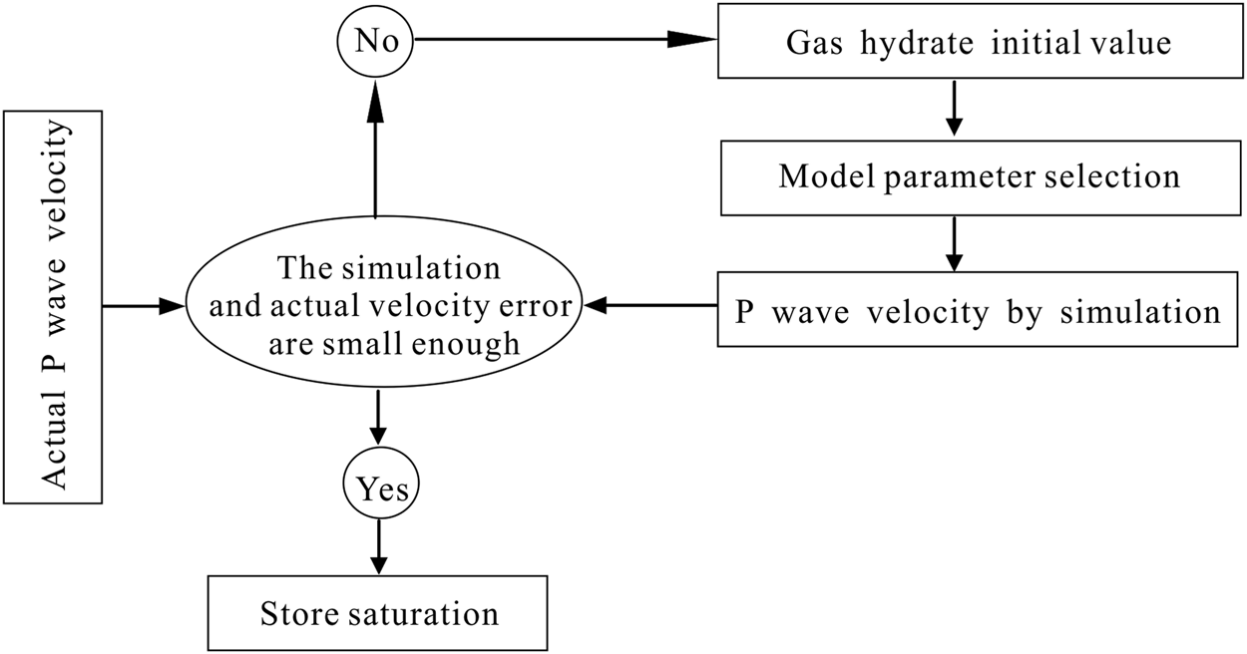
The flowchart for gas hydrate saturation inversion in pore-filling gas hydrate reservoir.

Figure 2 shows a flowchart for the inversion for the gas hydrate saturation using the P-wave velocity in the pore-filling reservoir. We set an initial gas hydrate saturation value, and then input the parameters of the formation rock matrix in the study area, such as porosity, density, bulk modulus, and shear modulus, and obtain the P-wave velocity under this saturation condition by use of equation (1). We compare the difference between the simulated P-wave velocity and the corresponding P-wave velocity observed in the acoustic log. If the difference between the values is within a preset permissible error range, then the gas hydrate saturation is considered to be the actual gas hydrate saturation of the reservoir. Otherwise, if the difference between the predicted and measured values is not within the permissible error range, then the modified saturation’s initial value is repeated until the error is met.

### Numerical simulation of fracture filling reservoir

#### Acoustic velocity forward simulation

**Figure 3:**
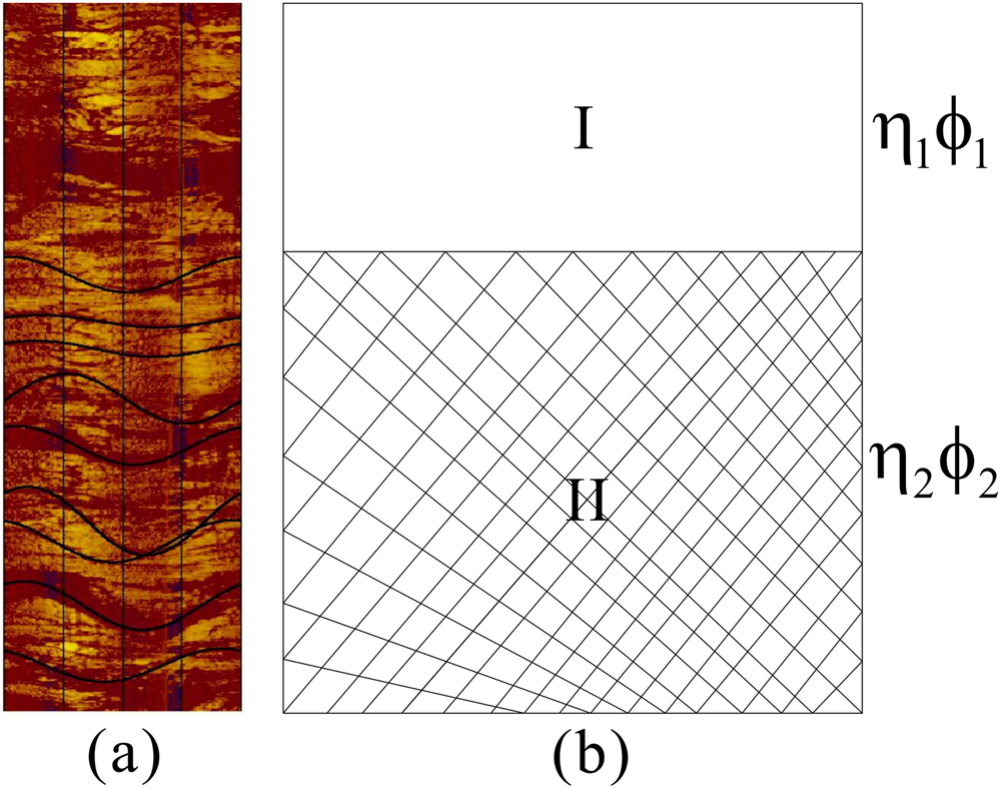
The ultrasonic imaging logging image and two component laminated media model.

Some drilling boreholes have implemented advanced ultrasonic imaging logging in scientific drilling project of QMP. This logging method can effectively identify formation fractures and analysis of fracture occurrence, and thus provides a basis for numerical simulation of the acoustic log in a fracture-filling gas hydrate reservoir. Figure 3(a) shows an ultrasonic imaging log of a gas hydrate sample in the study area. From the image, it can be seen that the cracks of this interval are well developed, mainly consist of high angle fractures, and the gas hydrates are mainly located in these high angle cracks. Figure 3(b) shows the two component laminated media model of the corresponding fractured reservoir, which is used to simulate the acoustic velocity characteristics of the gas hydrate in the cracks.

The TCLM model is composed of two component elements: I and II (Fig. 3(b)). The component I is the anisotropic fracture medium, and the cracks are 100% filled with gas hydrate. The component II is isotropic pore medium with fully saturated water in the pore. The volume fraction of the fracture medium in the component I is η_1_, fracture porosity is ϕ_1_, and assumes ϕ_1_ = 100. The volume fraction of the pore medium in the component II is η_1_, and saturated water porosity is ϕ_2_. The elastic parameters of the fractured gas hydrate reservoir can be expressed as follows:

5 $$\langle {\rm{M}}\rangle \equiv ({\eta }_{1}{{\rm{M}}}_{1}+{\eta }_{2}{{\rm{M}}}_{2})$$

6 $${\langle \frac{1}{{\rm{M}}}\rangle }^{-1}\equiv {(\frac{{{\rm{\eta }}}_{1}}{{{\rm{M}}}_{1}}+\frac{{{\rm{\eta }}}_{2}}{{{\rm{M}}}_{2}})}^{-1}$$

where M is the combination of arbitrary elastic parameters of the components I and II in Fig. 3(b). For the component I, the gas hydrate completely fills the cracks, and the elastic parameters can be replaced by the elastic parameters of pure gas hydrate. For component II, each elastic parameter can be calculated by the EMT model. The P- and S-wave phase velocities can be calculated from the following equations using the Lame constants *λ* and *μ* ^36^:

7 $${\rm{A}}=\langle \frac{4{\rm{K}}(\lambda +{\rm{\mu }})}{\lambda +2{\rm{\mu }}}\rangle +{\langle \frac{1}{\lambda +2{\rm{\mu }}}\rangle }^{-1}{\langle \frac{\lambda }{\lambda +2{\rm{\mu }}}\rangle }^{2}$$

8 $${\rm{C}}={\langle \frac{1}{\lambda +2{\rm{\mu }}}\rangle }^{-1}$$

9 $${\rm{F}}={\langle \frac{1}{\lambda +2{\rm{\mu }}}\rangle }^{-1}\langle \frac{\lambda }{\lambda +2{\rm{\mu }}}\rangle $$

10 $${\rm{L}}={\langle \frac{1}{{\rm{\mu }}}\rangle }^{-1}$$

11 $${\rm{N}}=\langle {\rm{\mu }}\rangle $$

12 $${\rm{\rho }}=\langle {\rm{\rho }}\rangle $$

13 $${\rm{Q}}=\sqrt{{[({\rm{A}}-{\rm{L}}){\sin }^{2}\phi -({\rm{C}}-{\rm{L}}){\cos }^{2}\phi ]}^{2}+4{({\rm{F}}+{\rm{L}})}^{2}{\sin }^{2}\phi {\cos }^{2}\phi }$$

14 $${{\rm{V}}}_{{\rm{P}}}={(\frac{{\rm{A}}{\sin }^{2}\phi +{\rm{C}}{\cos }^{2}\phi +{\rm{L}}+{\rm{Q}}}{2{\rm{\rho }}})}^{1/2}$$

15 $${{\rm{V}}}_{{\rm{S}}}^{{\rm{V}}}={(\frac{{\rm{A}}{\sin }^{2}\phi +C{\cos }^{2}\phi +{\rm{L}}-{\rm{Q}}}{2{\rm{\rho }}})}^{1/2}$$

16 $${{\rm{V}}}_{{\rm{S}}}^{{\rm{H}}}={(\frac{{\rm{N}}{\sin }^{2}\phi +{\rm{L}}{\cos }^{2}\phi }{{\rm{\rho }}})}^{1/2}$$

where φ is the propagation angle relative to vertical, V_P_ is the P-wave phase velocity, ${{\rm{V}}}_{{\rm{S}}}^{{\rm{h}}}$ is the horizontally polarized S-wave phase velocity, and ${{\rm{V}}}_{{\rm{S}}}^{{\rm{v}}}$ is the vertically polarized S-wave phase velocity. The P- and S-wave velocities calculated from equations (14), (15), and (16) are the phase velocities for the fractured gas hydrate reservoir. When the P- and S-wave phase velocities are subsequently used to invert for the gas hydrate saturation, they need to be converted into the transverse velocities. For a vertical borehole, modeling using an incidence angle of 0° represents wave propagation for a horizontal fracture and using an incidence angle of 90° represents wave propagation for a vertical fracture. The incidence angle is related to the angle of fracture, and the angle of incident angle reflects the dip angle of the fracture in the formation^26^. When the dip angle of the fracture is horizontal or vertical, the transverse velocity and phase velocity are consistent. When the fracture dip angle is any other angle, the phase velocity can be converted into the transverse velocity by use of Thomsen’s equation^37^.

#### Gas hydrate saturation inversion

**Figure 4:**
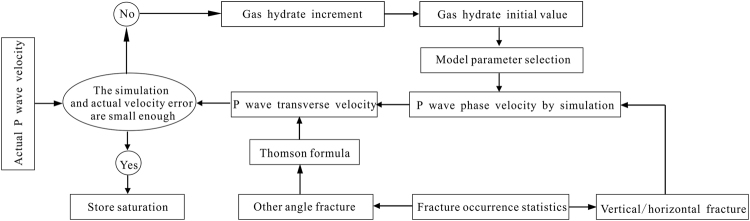
The flowchart of gas hydrate saturation inversion in fracture filling gas hydrate reservoir.

Figure 4 shows a flowchart for the inversion of gas hydrate saturation using the P-wave velocity in a fracture-filling gas hydrate reservoir. We set an initial gas hydrate saturation value, and then input the elastic parameters of the components I and II for the study area, such as porosity, density, bulk modulus, and shear modulus. We obtain a P-wave phase velocity under this saturation condition by use of equation (14). According to the statistics of fracture occurrence in the gas hydrate reservoir based on the ultrasonic imaging well logging image, it is necessary to determine whether the simulated P-wave phase velocity can be converted into the P-wave transverse velocity. Finally, we compare the difference between the simulated P-wave transverse velocity and the corresponding actual P-wave velocity determined from the acoustic log. If the difference is within the permissible error range, then the gas hydrate saturation is considered to be the actual gas hydrate saturation of the reservoir. Otherwise, if the difference between above two is not within the permissible error range, then the modified saturation’s initial value is repeated until the error is met.

## Results and Discussion

### Research on acoustic velocity and gas hydrate saturation from pore filling reservoir

**Figure 5:**
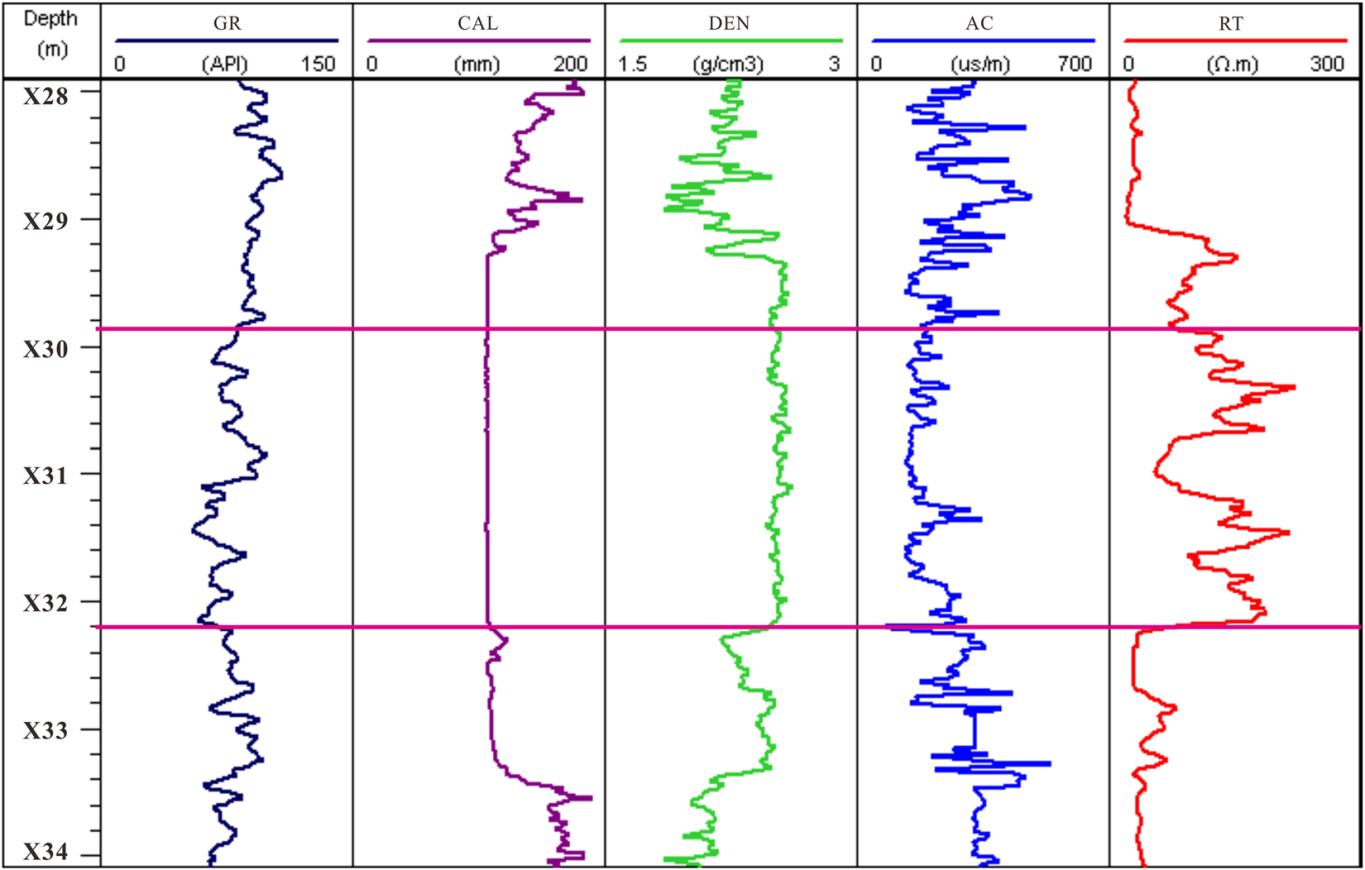
Conventional logging curve of the gas hydrate reservoir in hole DKXX-13, where GR is the total gamma-ray response, CAL is the caliper log, DEN is the density log, AC is the acoustic travel time, and RT is the resistivity log.

The hole DKXX-13 has implemented an integrated conventional well logging of the whole borehole section. The logging projects include natural gamma, well diameter (caliper), density, acoustic travel time, resistivity logging, and so on. Figure 5 shows the conventional well logging curves of the gas hydrate in a sample drilling interval (X29.8~X32.2 m). Due to the presence of gas hydrate, this interval has obvious responses in the resistivity and acoustic travel time logging curves, and shows the response characteristics of high resistivity (RT) and low acoustic travel time (AC), which are consistent with the logging response characteristics of gas hydrates in other permafrost regions around the world^14^. The natural gamma and density curves are mainly the reflection of strata lithology, which is mainly composed of argillaceous sandstone. At the same time, the well diameter curve can also be used as the effective parameter to identify the gas hydrate reservoir; the interval of the well diameter curve is relatively smooth and unchanging, and the other well log responses are not related to the borehole condition.

**Figure 6:**
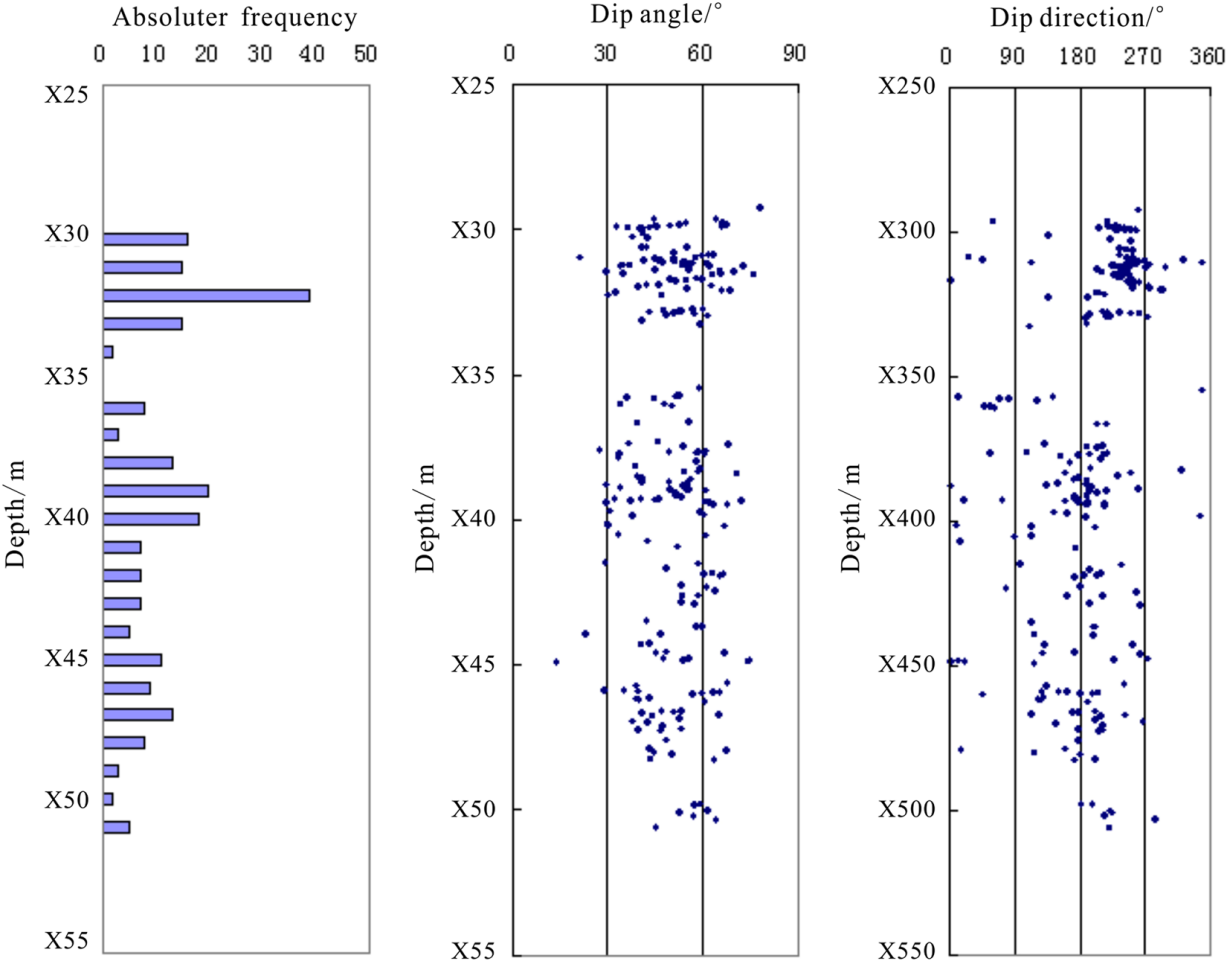
Statistical graph of the cracks in the borehole DKXX-13 in Qilian mountain permafrost.

In hole DKXX-13, we also carried out ultrasonic imaging logging during the drilling process. High quality borehole formation information was obtained, which can be used to analyze and study the development and occurrence distribution of fractures in the gas hydrate reservoir. In this paper, the cracks in the borehole are collated by means of the ultrasonic imaging logging image, and the characteristics of fracture occurrence with depth change are analyzed (Fig. 6). As shown for the depth range of X29.8~X32.2 m, 267 cracks are present, and the absolute frequency is 9.7 stripes/10 m, but we also see that the stratigraphic fracture in the whole borehole is not developed. In depth range from X29.8~X32.2 m of the reservoir, 32 cracks are collected, the absolute frequency is 13.7 stripe/10 m; the dip angles are mainly distributed over a range of 45°~75°, which indicated high angle fracturing^38^. The above analysis shows that the development of high angle fracture in gas hydrate reservoir of the hole DKXX-13 is inconsistent, which mainly reflects the gas hydrate filling in the pores of the borehole formation. The results agree with the gas hydrate sampling from field drilling.

#### Acoustic velocity characteristics analysis

By use of the acoustic velocities simulated by the EMT and TPT models, the formation porosity, *ϕ*, needs to be determined. Formation porosity is a key parameter for gas hydrate estimation, so appropriate log data should be selected to estimate the formation porosity of hole DKXX-13 in the study area. The log data that can be applied to determine the formation porosity include density, acoustic travel time, and resistivity logging. When the acoustic log is used to determine formation porosity, the calculated porosity needs to be corrected using the regional core data because gas hydrate is buried shallow in the study area. However, the core data is always insufficient, and it is difficult to determine the compaction correction coefficient, so the acoustic log is not suitable. The Archie formula should be used to calculate porosity for the resistivity log, and the Archie constants and formation water resistivity need to be known for the Archie formula^39^. However, the above two parameters are generally determined by some empirical equations, which will lead to significant estimation error. The density log is less affected than the resistivity and acoustic logs in gas hydrate reservoir, and can generally reflect the situation of formation porosity, so we select the density log to estimate the formation porosity in this paper.

The intensity of scattering gamma ray is measured for the density log, which reflects the electron density of the formation and the volume density of rock (${\rho }_{b}$). The porosity estimation by density log can be expressed as^40^:

17 $$\varphi =\frac{{\rho }_{ma}-{\rho }_{b}}{{\rho }_{ma}-{\rho }_{f}}$$

where ${\rho }_{ma}$ is the matrix density and ${\rho }_{ma}$ = 2.64 g/cm^3^ by use of the mineral component of the rock skeleton, ${\rho }_{f}$ is the fluid density, and ${\rho }_{f}$ ≈ 1.00 g/cm^3^ by use the density of pure water. Because of the high content of mud in the gas hydrate reservoir of hole DK XX-13, equation (17) needs to include a mud correction. So equation (17) can be corrected as follows^40^:

18 $$\varphi =\frac{{\rho }_{ma}-{\rho }_{b}}{{\rho }_{ma}-{\rho }_{f}}-{V}_{sh}\frac{{\rho }_{ma}-{\rho }_{sh}}{{\rho }_{ma}-{\rho }_{f}}$$

19 $${V}_{sh}=\frac{{2}^{GCUR\times SH}-1}{{2}^{GCUR}-1}$$

20 $$SH=\frac{GR-G{R}_{\min }}{G{R}_{\max }-G{R}_{\min }}$$

where ${V}_{sh}$ is the volume content of the mud, SH is the content index of the mud, ${\rho }_{sh}$ is the density of the mud, and *GR*, *GR*_min_, and *GR*_max_ are the values of the natural gamma log in the target layer, in the pure sandstone layer, and in pure mud layer, respectively, and *GCUR* is the Hilchie index^41^.

**Figure 7:**
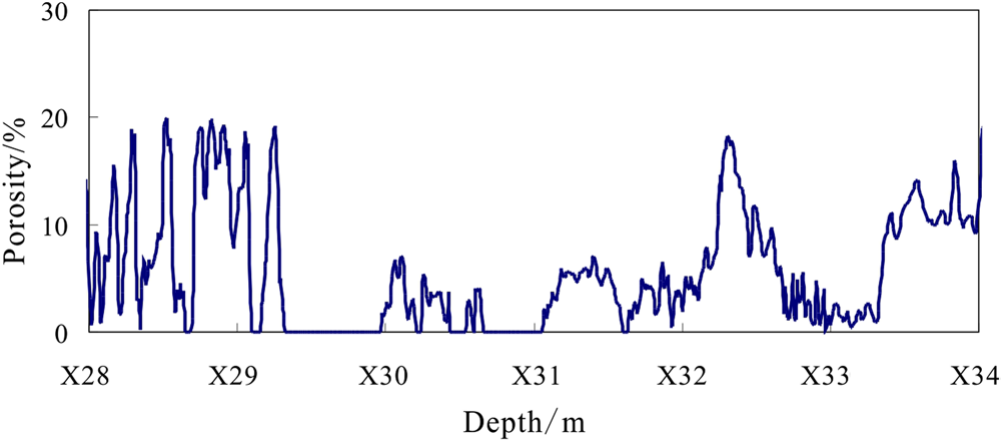
Calculated formation porosity by density log in depths of X28.0~X34.0 m of hole DKXX-13.

Equation (18) is used to calculate the formation porosity in depths of X28.0~X34.0 m of hole DKXX-13 (Fig. 7). The mud content in the depth ranges of X29.3~X30.0 m and X30.7~X31.1 m are quite high, so the porosities of those intervals are 0, which indicates that the lithology is mudstone. Because of the effect of borehole enlargement in the depth ranges of X28.0~X29.3 m and X33.4~X34.0 m, the calculated porosities of those intervals are too large. In the depth range of X29.8~X32.2 m, the calculated porosities vary from 1.0~8.0%, the average value is 4.0%, and standard error is 0.1%. According to the porosity test results of different granulometric class sandstone, the test porosities vary from 2.2~5.1%, the average value is 3.5%^42^. The calculated porosities are basically the same as the core porosity test results. The result indicates that the formation porosity of the gas hydrate reservoir is quite low, which means that the lithology of the reservoir in hole DKXX-13 is more compact.

**Figure 8:**
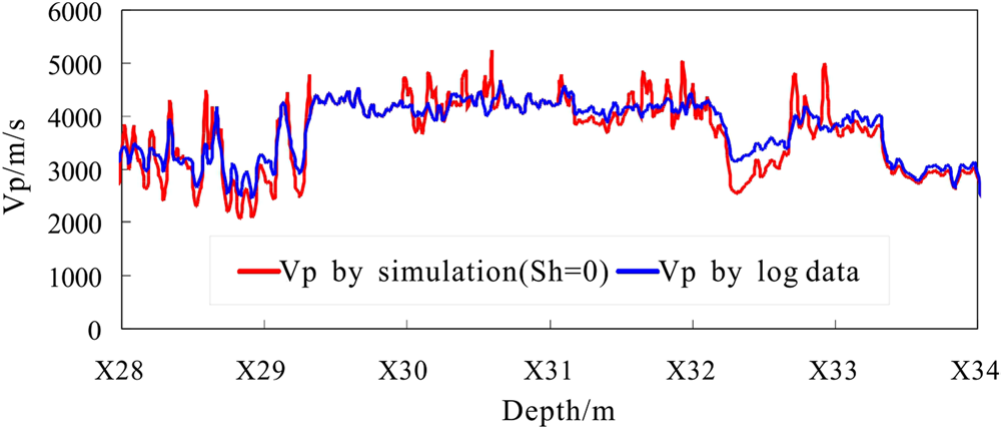
Forward simulation P-wave velocity from the EMT model in hole DKXX-13 compared to the observed P-wave velocity.

**Figure 9:**
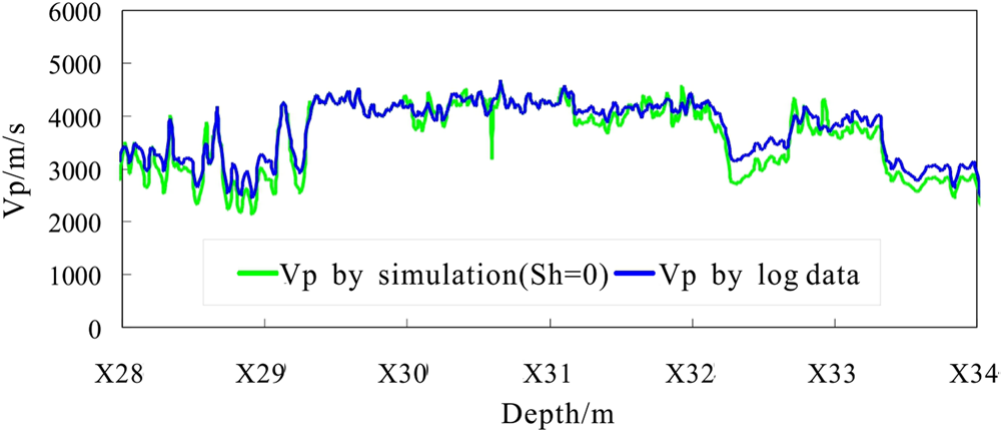
Forward simulating P-wave velocity by the TPT model in hole DKXX-13.

According to the natural gamma log data, the shale content of borehole formation in hole DKXX-13 is high, and large parts of the borehole intervals are mudstone. Therefore, only the non-mudstone formation is carried out acoustic velocity characteristics simulation by use of the EMT model and TPT model. If we assume that the borehole formation is water saturated (i.e., the gas hydrate saturation *S*_*h*_ = 0), then the P-wave velocities in the depth range of X28.0~X34.0 m are simulated by the EMT model (Fig. 8). Meanwhile, the simulation results of the TPT model are comparable (Fig. 9). Some values of the parameters in above two velocity models are shown in Tables 2 and 3.

**Table 2:** Values of the parameters of the TPT model.

Parameters	Values	References
*C*_*s*_	2.7 × 10^−11^ *Pa* ^−1^	51
*C*_*h*_	1.79 × 10^−10^ *Pa* ^−1^	43
*C*_*w*_	4.79 × 10^−10^ *Pa* ^−1^	22
*C*_*g*_	4.24 × 10^−8^ *Pa* ^−1^	52

**Table 3:** Values of elastic parameters of rock components^22,24^.

Components	*K* (GPa)	*μ* (GPa)	*ρ* (g/cm^3^)
Clay	20.90	6.60	2.58
Calcite	76.80	32.00	2.71
Quartz	38.00	44.00	2.65
Gas hydrate	7.90	3.30	0.90
Water	2.50	0	1.00

From Fig. 8, the P-wave velocity curve for the water-saturated formation obtained from the EMT model and the actual curve of the P-wave velocity log are almost coincident in the depth ranges of X28.0~X29.8 m and X32.2~X34.0 m (the non-gas hydrate interval), and the two curves coincide in the depth range of X33.5~X34.0 m. We can be seen that the EMT model and the parameters used can be used to reliably analyze the velocity characteristics of the pore-filling gas hydrate reservoir in the study area.

From Fig. 9, the results of the P-wave velocity for the water-saturated formation using the TPT model are consistently less than the values obtained by the EMT model. In the non-gas hydrate interval, the P-wave velocity curve for the water-saturated formation from the TPT model and the actual P-wave velocity log are almost coincident, but do not coincide, which can not reflect the actual saturation water formation situation. The P-wave velocity curve by the TPT model has some deviation from the actual P-wave velocity curve. Therefore, the EMT model can be better used to analyze the P-wave velocity and gas hydrate saturation characteristics of the pore filling gas hydrate reservoir in the study area.

The difference between the actual log P-wave velocity and saturated water P-wave velocity reflects gas hydrate or free gas saturation concentration, and can be used to qualitatively identify gas hydrate reservoir^22,43^. The method for the identification of gas hydrate or free gas in the formation by using the simulated saturated water P velocity curve is as follows:

If the actual log P-wave velocity is higher than that for a water-saturated formation, then the formation interval may contain gas hydrate;

If the actual log P-wave velocity is lower than that for a water-saturated formation, then the formation interval may contain free gas.

From Fig. 8, in the interval X29.8 to X32.2 m, the multiple curves (X30.0~X30.2 m, X30.3~X30.4 m, X31.1~X31.6 m, X31.7~X31.9 m, and X32.0~X32.2 m) show that actual log P-wave velocity is greater than for the water-saturated P velocity, which reflects the presence of gas hydrate. In the intervals from X28.0~X29.3 m, X32.3~X32.7 m, and X32.8~X33.4 m, the actual log P-wave velocity also shows values greater than the water-saturated P velocity, but the resistivity log curves show no increase in resistivity values in that segment of Fig. 5, which can be inferred that these anomalies may be caused by gas hydrate melting.

#### Gas hydrate saturation estimation

**Figure 10:**
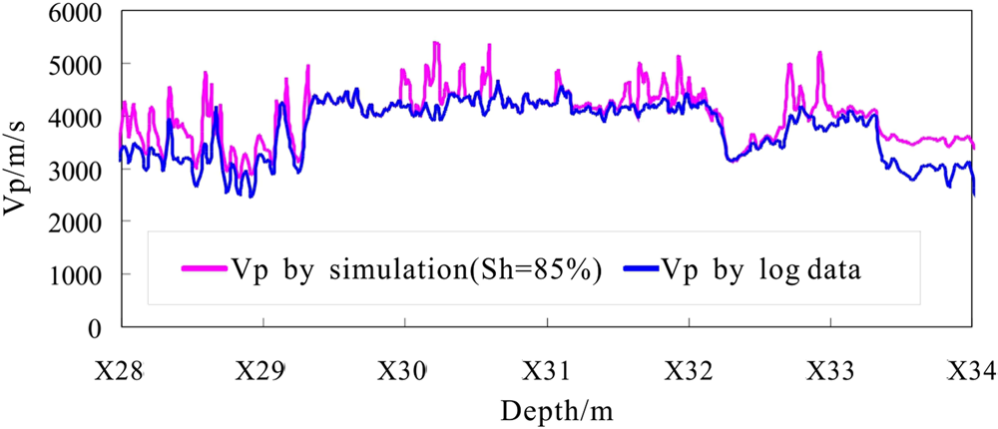
Forward simulation P-wave velocity for depths of X28.0~X34.0 m of hole DKXX-13 compared to the observed P-wave velocity from the logging data.

When we estimate the gas hydrate saturation by inversion using the EMT model, we can set a relatively large initial guess for the gas hydrate saturation, and use forward modeling of the P-wave velocity curve to determine the scope of the change of the gas hydrate saturation Then the scope of the search for a fit can be reduced to reduce the inversion workload. From Fig. 10, with the increase of gas hydrate saturation (*S*_*h*_), the simulated P-wave velocity increases, and when ${S}_{h}=85.0 \%$, the whole curve of P-wave velocity by simulated coincides with the actual P-wave velocity curve from the acoustic login the depth range X28.0~X34.0 m of hole DKXX-13, which reflects the range of gas hydrate saturation of 0~85.0%.

**Figure 11:**
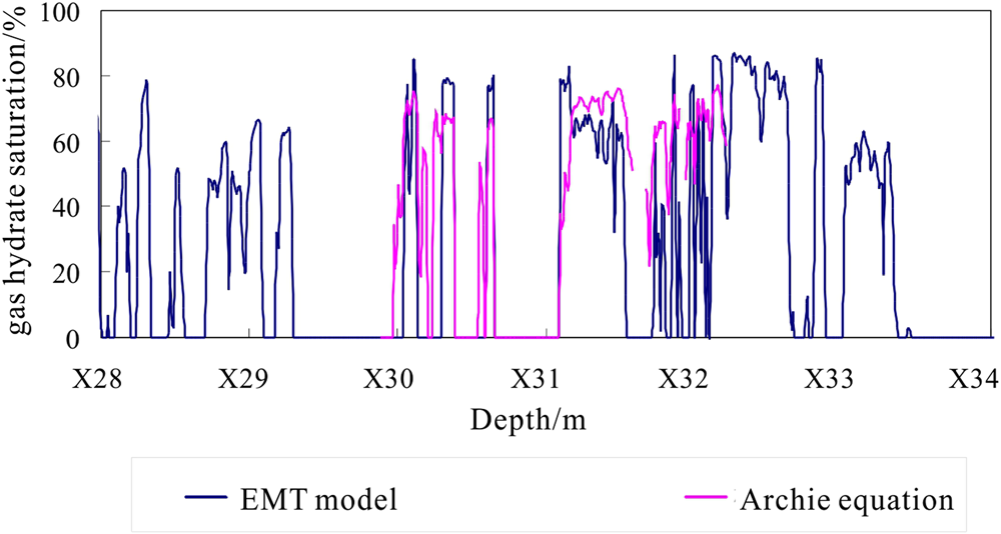
Gas hydrate saturation inversion values for depths of X28.0~X34.0 m in hole DKXX-13.

The gas hydrate saturation for depths of X28.0~X34.0 m in hole DKXX-13 is inverted using the EMT model (Fig. 11). At the same time, in order to compare and analyze the inversion results, gas hydrate saturation is also estimated from the resistivity logging using the Archie equation^39^. From the blue curve, the estimated gas hydrate saturation for depths of X28.0~X34.0 m is relatively large. Because the lithology of the borehole formation is relatively dense, the porosity of the formation is small, and so the volume percentage of gas hydrate in the pore of the borehole formation is large enough to be detected. In the intervals of abnormal gas hydrate concentrations (X28.0~X29.3 m, X32.3~X32.7 m, X32.8~X33.4 m), the range of gas hydrate saturation is 3.0~86.0%, the average value is 52.3%, and standard error is 1.7%. The inversion results show existing gas hydrate occurrences for depths of X30.0~X30.2 m, X30.3~X30.4 m, X31.1~X31.6 m, X31.7~X31.9 m, and X32.0~X32.2 m. The range of gas hydrate saturation is 13.0~85.0%, the average value is 61.9%, and standard error is 1.9%, which reflects the relatively high gas hydrate saturation.

From the pink curve in Fig. 11, the gas hydrate saturation curve from the EMT model and the saturation curve by the density equation are comparable, although there are some differences in the peak values of the two curves. The estimation results indicate the presence of gas hydrate at depths of X29.8~X30.2 m, X30.3~X30.4 m, X30.5~X30.7 m, X31.1~X31.6 m, X31.7~X31.9 m, and X32.0~X32.2 m; the range of gas hydrate saturation is 18.0~77.0%, the average value is 61.6%, and standard error is 1.3%. The estimation results from the Archie equation show that while the peak value of gas hydrate saturation is lower than the EMT model, the average value of gas hydrate saturation is basically the same. What is more, the actual core test data for gas hydrate bearing sediments of pore-filling reservoir in study area show the gas hydrate saturations range from 52.9~61.8%, the average value is 58.8%^44^. The above study results are basically in accordance with the calculated result made by the EMT model. Thus the estimation of the gas hydrate saturation in pore-filling gas hydrate reservoir is reliable using the EMT model.

### Research on acoustic velocity and gas hydrate saturation from fracture filling reservoir

**Figure 12:**
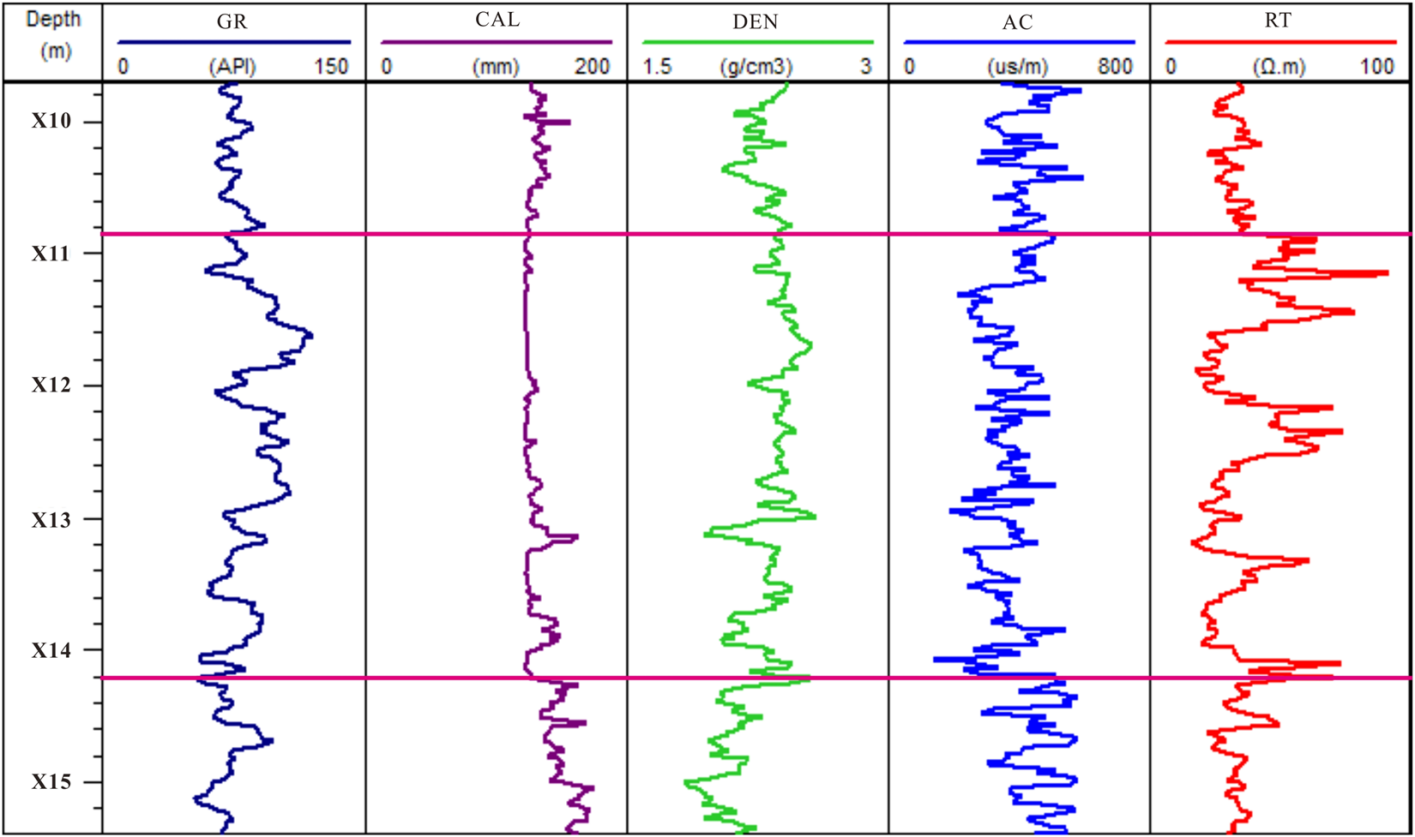
Conventional logging curve of the gas hydrate reservoir in hole DKXX-19. GR is the gamma ray response, CAL is the caliper log, DEN is the density log, AC is the acoustic travel time, and RT is the resistivity log.

The gas hydrate samples were acquired for depths of X10.9~X14.2 m in hole DKXX-19; the total thickness of the occurrence of gas hydrate is 33.7 m (Fig. 12). Where the gas hydrate occurs, conventional logging curves also show the response characteristics of high resistivity and low acoustic travel time. For depths of X11.4~X11.9 m, X12.6~X12.7 m, and X12.8~X12.9 m, conventional logging curves show the response characteristics of low resistivity and high acoustic travel time, which reflect a lack of gas hydrate in these borehole formations. For the caliper curve, multiple intervals display diameter fluctuation variation in the occurrence of gas hydrate, which reflect the high degree of fracture development in hole DKXX-19.

**Figure 13:**
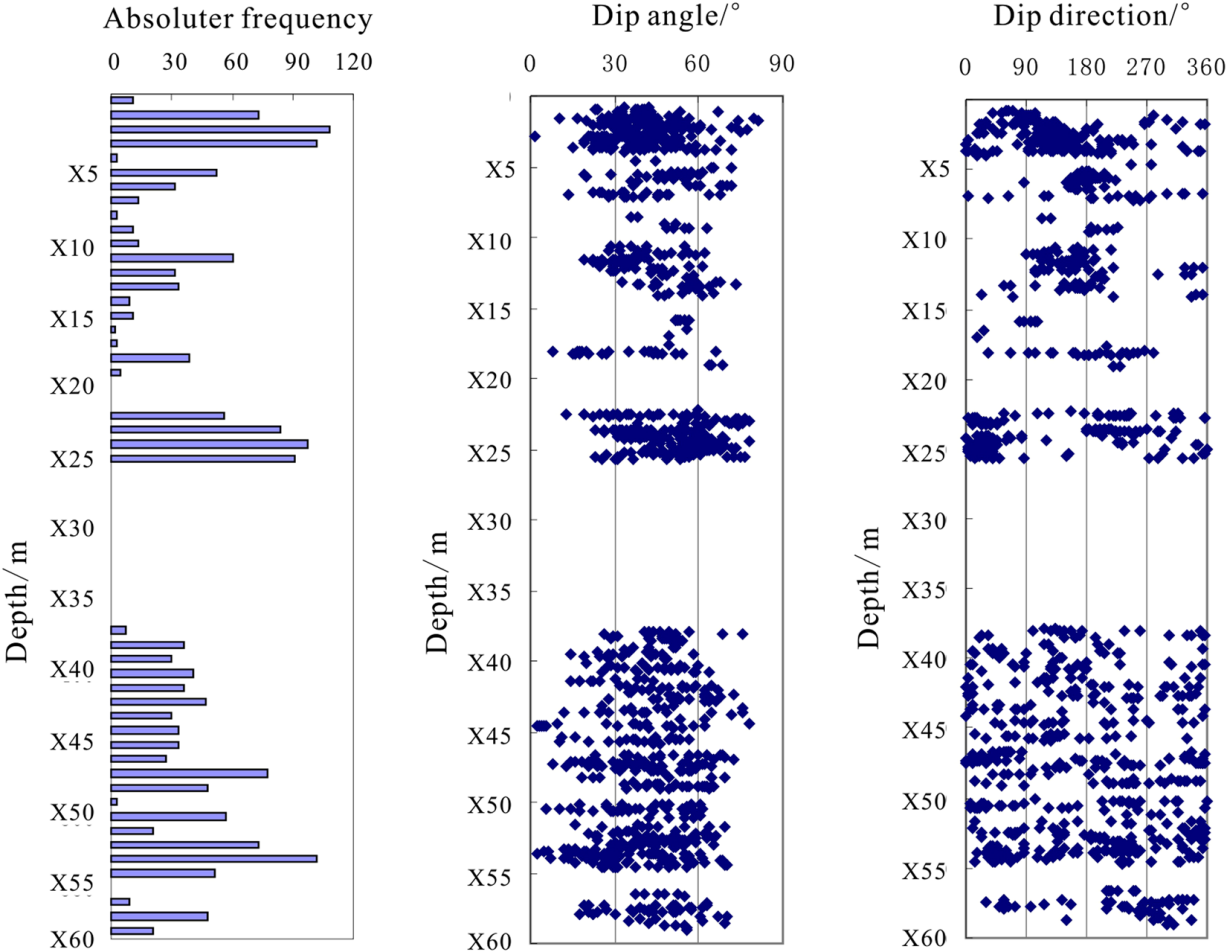
Statistical graph of the cracks of hole DKXX-19 in Qilian mountain permafrost.

Ultrasonic imaging logging work was also carried out in hole DKXX-19 during the drilling process, and the characteristics of fracture occurrence with depth change are also analyzed (Fig. 13). As shown in all sections, 1986 cracks are present, and the absolute frequency is 33.1 stripes/10 m, which shows that the stratigraphic fracture in the whole borehole is quite developed. For depths of X10.9~X14.2 m in the gas hydrate reservoir, 157 cracks are collected, the absolute frequency is 46.6 stripes/10 m; the maximum absolute frequency is 64 stripes/10 m, and the dip angles are mainly distributed in a range of 50°~70°, which indicates high angle fractures. The foregoing analysis shows that the development of high angle fractures in the gas hydrate reservoir of the hole DKXX-19 is relatively predominant, which reflects gas hydrate filling in the cracks of the borehole formation. The results agree with the response characteristics of conventional logging curves and the gas hydrate sampling from field drilling.

#### Acoustic velocity characteristics analysis

**Figure 14:**
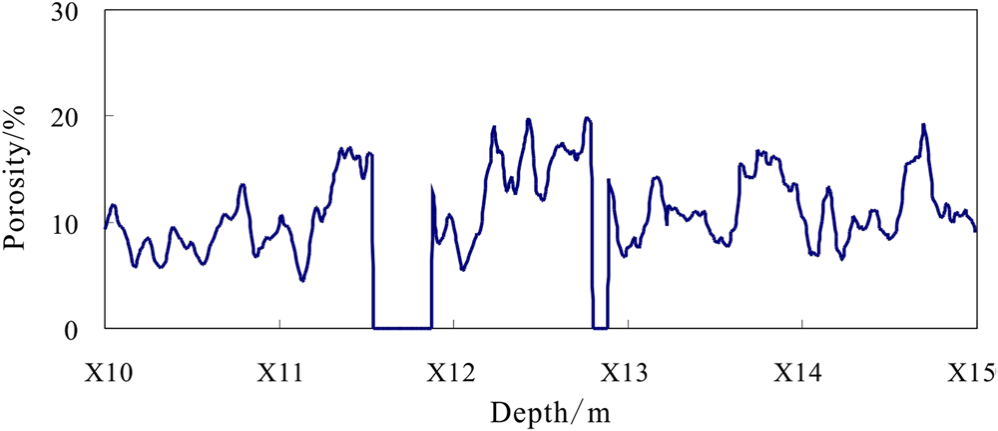
Calculated formation porosity by density log in depths of X10.0~X15.0 m in hole DKXX-19.

Equation (18) is used to calculate the formation porosity for depths of X10.0~X15.0 m in hole DKXX-19 (Fig. 14). The calculated porosities for depths of X11.5~X11.9 m and X12.8~X12.9 m are 0, which indicate that the lithologies in those intervals are most likely mudstone. For depths of X10.9~X14.2 m, the calculated porosities vary in the range of 5.0~20.0%, the average value is11.9%, and standard error is 0.2%. According to the core test results for the reservoir properties in Qilian mountain permafrost, the porosities range from 4.2~13.3%, the average value is10.6%, the calculated porosities is basically in accordance with the core test results^45^. The result indicates that the formation porosity of the gas hydrate reservoir is quite high. Because the density of gas hydrate is similar to that of the formation pore water, the calculated porosity from the density log can be approximated as the total porosity of the formation, and will include the fracture porosity and the porosity of pores saturated with water^46,47^.

For the fractured gas hydrate reservoir, the P-wave and S-wave velocities simulated by the TCLM model are affected by the changes in the gas hydrate content and the fracture dip angles in the cracks. Therefore, it is necessary to analyze the relations between these two parameters and the P-wave and S-wave velocities, so as to better carry out the relevant analysis.

**Figure 15:**
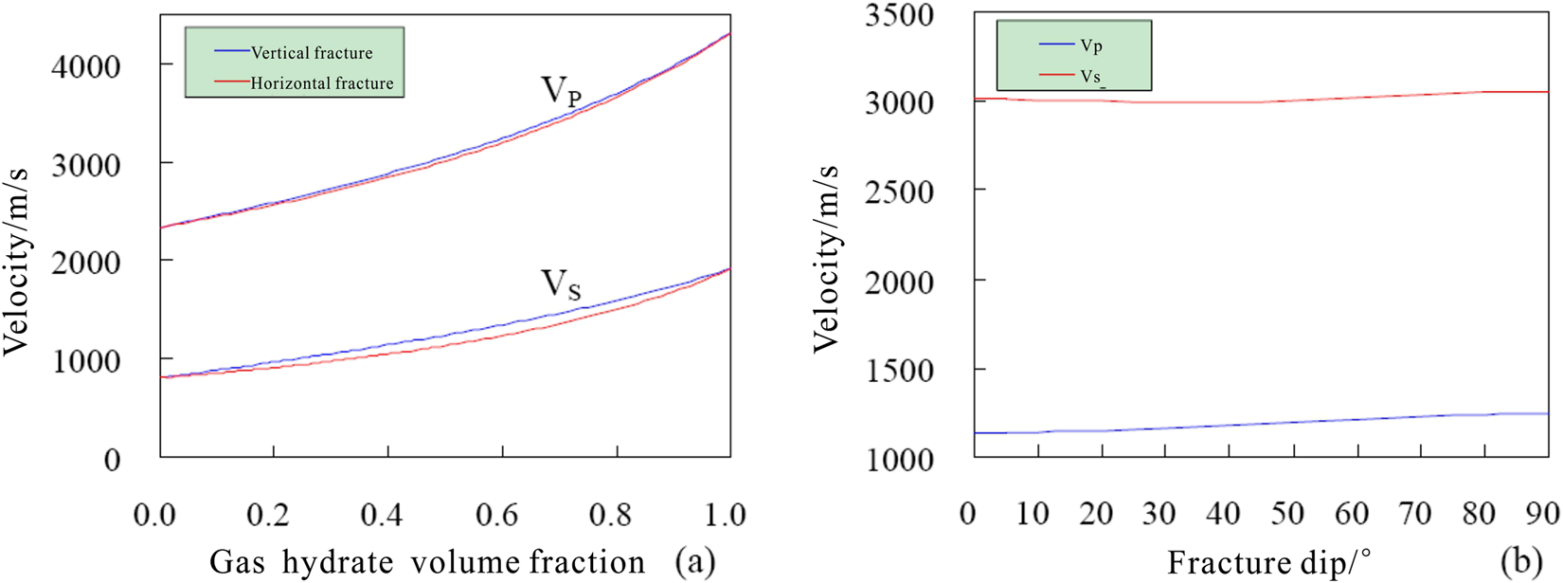
P-wave and S-wave velocities versus (**a**) volume fraction of gas hydrate and (**b**) fracture dip angle.

In order to determine the effect of the gas hydrate content on the P-wave and S-wave velocities, we set the formation porosity *ϕ* = 30.0%, the fracture dip angle is set to vertical and horizontal, respectively, and the volume and parameter values of each component of the formation rock skeleton are shown in Table 3. The P-wave velocity (*V*_*P*_) and horizontally polarized S-wave velocity (${V}_{S}^{H}$) versus volume fraction of gas hydrate are simulated using the TCLM model (Fig. 15(a)). When ${V}_{h}=0$, which indicates that there is no hydrate in the fracture, the simulated velocities represent the saturated water situation of the formation. When ${V}_{h}=1$, which indicates that the model pore space is completely filled with gas hydrate in the fracture, then the simulated velocities represent the parameter values of pure gas hydrate. For the vertical and horizontal fractures, when the volume fraction of gas hydrate increases, *V*_*P*_ and ${V}_{S}^{H}$ increase rapidly. For the same volume fraction of gas hydrate, when the fracture dip angle changes from horizontal to vertical, the ${V}_{S}^{H}$ increases rapidly, and the two S-wave velocity curves are clearly separated; the ${V}_{S}^{H}$ increases slowly, and the two P-wave velocity curves are effectively coincident.

To analyze the effect of fracture dip angle on the P-wave and S-wave velocities, we set the formation porosity $\varphi =30.0 \%$, and the volume fraction of gas hydrate ${V}_{h}=0.5$. Then the *V*_*P*_ and ${V}_{S}^{H}$ versus fracture dip angle are simulated using the TCLM model (Fig. 15(b)). When the fracture dip angle is small (<40°), *V*_*P*_ and ${V}_{S}^{H}$ change slowly. When the fracture dip angle is greater than 40°, the *V*_*P*_ and ${V}_{S}^{H}$ increase slowly with increasing fracture dip angle, and the range of velocity increase is quite little.

Through the above analysis, we see that when $\varphi  < 30.0 \%$, the change of volume fraction of gas hydrate filling in the fracture has great influence on the P-wave and S-wave velocities, whereas the change in the fracture dip angle has little influence on the velocities. Given that the average value of the formation porosity for depths of X10.0~X15.0 m in hole DKXX-19 is 11.9%, and the fracture dip angles vary mainly from 50°~70°, the fracture dip angle of the borehole formation can be assumed to be vertical. Therefore, the simulated P-wave phase velocity of hole DKXX-19 can be converted directly to the P-wave transverse velocity.

**Figure 16:**
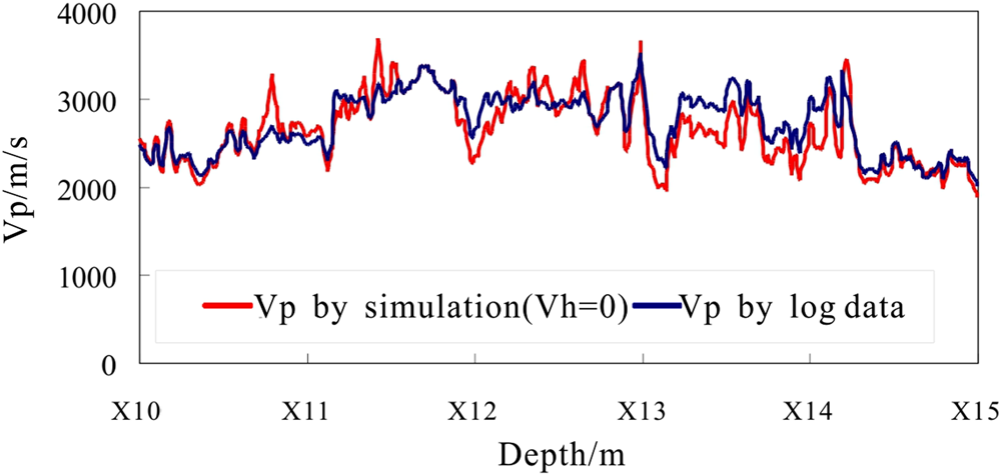
Forward simulation P-wave velocity using the TCLM model in hole DKXX-19, compared with the observed P-wave velocity determined from the acoustic log.

Assuming ${S}_{h}=0$, the P-wave velocity is simulated by the TCLM model for depths of X10.0 ~X15. 0 m in hole DKXX-19 (Fig. 16). The P-wave velocity curve for a water-saturated formation using the TCLM model and the actual curve of the P velocity log are almost coincident for depths of X10.0~X10.9 m and X14.2~X15.0 m (i.e., the intervals with no gas hydrate), and the two curves coincide for depths of X10.6~X10.8 m and X14.6~X14.7 m. It can be seen that the TCLM model and parameter settings can be used to reliably analyze the velocity characteristics of the fracture filling gas hydrate reservoir in the study area.

From Fig. 16, in the interval of X10.9 to X14.2 m, the multiple curves (X11.1~X11.3 m, X11.9~X12.2 m, X12.5~X12.6 m, X12.7~X12.8 m and X12.9~X14.2 m) show that actual log P-wave velocity is increasing than saturated water P velocity, which reflects the above segment existing gas hydrate. In the interval of X10.1~X10.6 m, X14.3~X14.6 m and X14.7~X15.0 m, actual log P-wave velocity also shows increasing than saturated water P velocity, but the resistivity log curves show no increasing in resistivity values in the above segment of Fig. 12, which can be inferred that these anomalies caused by gas hydrate melting.

#### Gas hydrate saturation estimation

**Figure 17:**
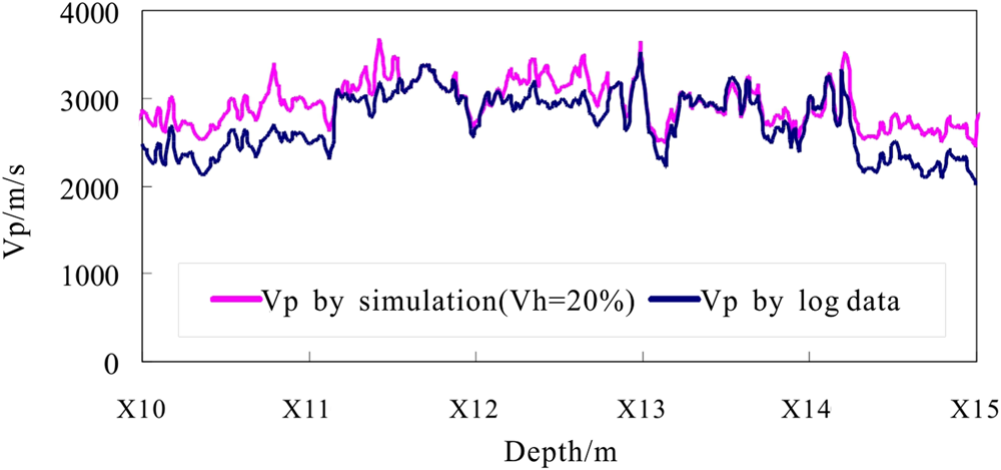
Forward simulatiion P-wave velocity in depths of X10.0~X15.0 m of hole DKXX-19, compared with the P-wave velocity as determined from the acoustic log.

When we obtain gas hydrate saturations by inversion using the TCLM model, the set inversion parameter is *V*_*h*_, and the conversion relation between *V*_*h*_ and *S*_*h*_ is *S*_*h*_ = *V*_*h*_/*ϕ*, where $\varphi$ is the total porosity by equation (18). Therefore, if we set a relatively large initial *V*_*h*_, and perform forward modeling of the P-wave velocity curve to determine the scope of the change of the gas hydrate saturation, then the search scope can be reduced to reduce the inversion workload. From Fig. 17, when *V*_*h*_ increases, the simulated P-wave velocity increases, and when ${V}_{h}=20 \%$, the whole curve of P-wave velocity by simulated is located at the top of the actual P-wave velocity curve as determined from the acoustic log, which reflects the range of gas hydrate saturation is 0~20% in depths of X10.0~X15.0 m of hole DKXX-19.

**Figure 18:**
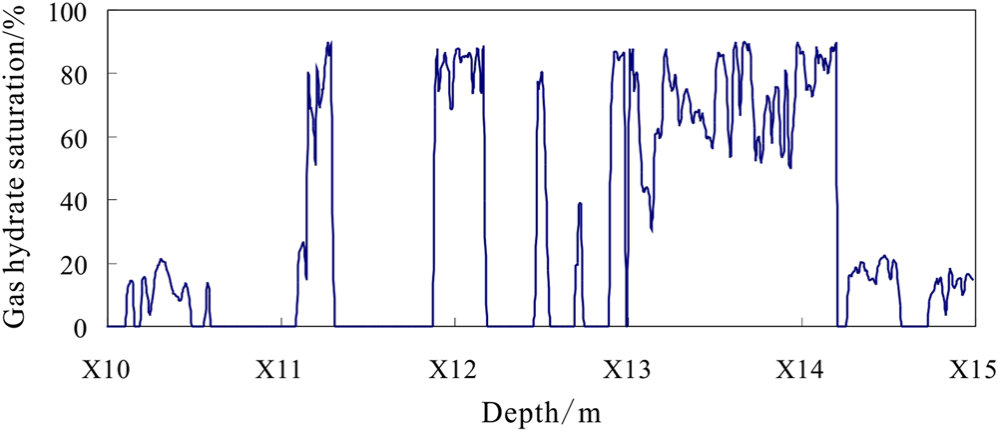
Estimated gas hydrate concentrations for depths of X10.0~X15.0 m in hole DKXX-19.

The gas hydrate saturation for depths of X10.0~X15.0 m of hole DKXX-19 is detemined using the TCLM model (Fig. 18). In the intervals of abnormal gas hydrate (X10.1~X10.6 m, X14.3~X14.6 m, and X14.7~X15.0 m), the range of gas hydrate saturation is 3.4~22.5%, the average value is 14.5%, and standard error is 0.5%. The results show the presence of significant gas hydrate at depths of X11.1~X11.3 m, X11.9~X12.2 m, X12.5~X12.6 m, X12.7~X12.8 m, and X12.9~X14.2 m, the range of gas hydrate saturation is 14.1~89.9%, the average value is 69.4%, and standard error is 1.3%, which reflects relatively high gas hydrate saturation in hole DKXX-19.

The actual core test data for gas hydrate bearing sediments of fracture-filling reservoir in study area show the gas hydrate saturations range from 72.3~75.8%, the average value is 73.5%^44^. What is more, the rang of simulated gas hydrate saturation is 0~80.0% based on porosity of 7.0% and 10.0% by Liu *et al*.^48^. The above previous study results are basically in accordance with the calculated result made by the TCLM model. This indicates that using the TCLM model to estimate gas hydrate saturation of fracture-filling reservoir is available.

Through the simulation results in this paper, in unfractured gas hydrate bearing sands in the QMP, China, an advantage of the EMT model by Helgerud *et al*. over the TPT model by Domenico is that whereas the EMT model accurately predicts P-wave velocities. In fracture-filling gas hydrate reservoirs, the method of combining ultrasonic log data with the TCLM model can be effectively used to evaluate gas hydrate saturations of gas hydrate formation. When gas hydrates usually fill the cracks in formation, the dip angle of the cracks has a certain influence on the estimation of gas hydrate saturations by the TCLM model. In this paper, fracture dip angles obtained by gas hydrate drilling borehole are steep and assuming the cracks are vertical, so a gas hydrate saturation error associated with the fracture dip angle error may exist. The fracture dip angle is related to the regional tectonic environment and presents the orientation characteristic, indicating that the fracture-filling gas hydrate is different from the pore-filling gas hydrate, and gas hydrate bearing sediments have anisotropic properties. For the gas hydrate saturation estimated by the EMT model, we have referred some empirical parameters from permafrost zone of other countries, so the inversion results may have some limitations. Although there are some empirical parameters by using above methods, the methods proposed in this paper are very necessary for the study of gas hydrate in this area. The research results could provide important reference for gas hydrate reservoir logging evaluation and seismic exploration in the QMP, China.

## Conclusions

1. The combination of ultrasonic imaging logging and drilling core data can be used to identify the filling type of gas hydrate reservoir in Qilian mountain permafrost. The hole DKXX-13 illustrates the typical pore filling gas hydrate reservoir, and the hole DKXX-19 represents the typical fracture filling gas hydrate reservoir.
2. Using the EMT and TPT models of pore filling gas hydrate reservoirs, the characteristics of the acoustic velocity for hole DKXX-13 are analyzed. The P-wave velocity simulated by the EMT model is more consistent with the actual log data than the TPT model.
3. By comparing the simulated P velocity and the velocity from the actual log data, the occurrence of gas hydrate is identified to occur at depths of X30.0~X30.2 m, X30.3~X30.4 m, X31.1~X31.6 m, X31.7~X31.9 m, and X32.0~X32.2 m of hole DKXX-13. The inversion estimation results by the EMT model suggest that the range of gas hydrate saturation is 13.0~85.0%, and the average value of the gas hydrate saturation is 61.9%, which is largely in accordance with the results derived using the standard Archie equation.
4. Using the TCLM model of a fracture-filling gas hydrate reservoir, the characteristics of the acoustic velocity of hole DKXX-19 are analyzed. The change in the volume fraction of gas hydrate filling the fractures greatly influences the P-wave and S-wave velocities, but the change in fracture dip angle has much less effect on the velocities. The P-wave phase velocity simulated using the TCLM model can be transformed directly into the P-wave transverse velocity.
5. The occurrence of gas hydrate is estimated to occur at depths of X11.1~X11.3 m, X11.9~X12.2 m, X12.5~X12.6 m, X12.7~X12.8 m and X12.9~X14.2 m of hole DKXX-19.The inversion estimation results using the TCLM model estimate that the range of gas hydrate saturation is 14.1~89.9%, and the average value of the gas hydrate saturation is 69.4%, which is largely in accordance with the core test results.
